# Deep Reinforcement Learning for Autonomous Driving with an Auxiliary Actor Discriminator

**DOI:** 10.3390/s24020700

**Published:** 2024-01-22

**Authors:** Qiming Gao, Fangle Chang, Jiahong Yang, Yu Tao, Longhua Ma, Hongye Su

**Affiliations:** 1 Ningbo Innovation Center, Zhejiang University, Ningbo 315100, China; 22032106@zju.edu.cn (Q.G.); 22360417@zju.edu.cn (J.Y.); taoyu@zju.edu.cn (Y.T.); lhma_zju@zju.edu.cn (L.M.); hysu69@zju.edu.cn (H.S.); 2 State Key Laboratory of Fluid Power and Mechatronic Systems, Zhejiang University, Hangzhou 310027, China; 3 Polytechnic Institute, Zhejiang University, Hangzhou 310013, China; 4 Institute of Intelligent Automation, NingboTech University, Ningbo 315100, China

**Keywords:** autonomous driving, reinforcement learning (RL), auxiliary actor discriminator (AAD), heuristic knowledge (HK)

## Abstract

In the research of robot systems, path planning and obstacle avoidance are important research directions, especially in unknown dynamic environments where flexibility and rapid decision makings are required. In this paper, a state attention network (SAN) was developed to extract features to represent the interaction between an intelligent robot and its obstacles. An auxiliary actor discriminator (AAD) was developed to calculate the probability of a collision. Goal-directed and gap-based navigation strategies were proposed to guide robotic exploration. The proposed policy was trained through simulated scenarios and updated by the Soft Actor-Critic (SAC) algorithm. The robot executed the action depending on the AAD output. Heuristic knowledge (HK) was developed to prevent blind exploration of the robot. Compared to other methods, adopting our approach in robot systems can help robots converge towards an optimal action strategy. Furthermore, it enables them to explore paths in unknown environments with fewer moving steps (showing a decrease of 33.9%) and achieve higher average rewards (showning an increase of 29.15%).

## 1. Introduction

In recent years, driverless technology has caused much attention with the development of artificial intelligence and information perception [1]. Avoiding obstacles efficiently is important for intelligent robots to explore an accurate route in unknown environments, which include sweeping robots, mining robots, and rescue robots [2]. Commonly used obstacle avoidance methods include the gap-based algorithm [3], artificial potential field algorithm [4], velocity obstacle algorithm, and neural network algorithms [5]. The gap-based algorithms are based on classical reactive navigation and show good performance in areas with dense obstacles by sensing real-time environment information. One problem is that they may cause unreasonable deviation toward free areas, increasing the total distance and time to execute the mission. The artificial potential field algorithm is commonly developed for dynamic obstacle avoidance, and always has a local minimum. It calculates the resultant virtual force to facilitate the real-time control of the intelligent robot’s control layer. The local minimum causes the robot to fall into a local oscillation and fail to find a global optimal solution, and many studies solve the problem by performing a random walk mechanism [6] and by using a navigation function [7]. Bounini et al. solved the problem by adding some extra repulsive potential, inspired by pouring a liquid with high pressure [4].

Reciprocal velocity obstacles (RVO) and its subsequent products aim to formulate a potential collision area for a moving obstacle using the relative velocity and position [8]. In these methods, a velocity outside this area is chosen for the robot to complete the collision avoidance task, and a distributed real-time multiple vehicle collision avoidance (MVCA) algorithm is proposed by extending the reciprocal n-body collision avoidance method [9]. However, it requires a perfect sensing situation in the approaches [10]. Fuzzy algorithms do not require an exact mathematical model and perform well to overcome local extreme value problems. One needs to define all the regular behaviors for a fuzzy algorithm [11]. The application of artificial intelligence in obstacle avoidance has received a lot of attention in recent years. Jiang et al. [2] proposed the Deep Q-learning (DQL) algorithm to achieve obstacle avoidance in unknown environments for navigation. Zhang et al. [12] proposed a novel adaptive obstacle avoidance algorithm for USVs, based on the Sarsa on-policy Reinforcement Learning (RL) algorithm. Generally, neural network algorithms need to be trained with a big number of obstacle avoidance data.

Intelligent robots need detailed environment information to autonomously plan a path. In path planning, one common method is using global environmental data, and the other one is only using local environmental information [7]. Using global environmental data, predefined maps are usually constructed to describe the geometric information of the environment and are constantly updated during the navigation process, which requires a lot of memory and computational resources [13]. The whole process needs a model of the entire gridded/topology map and includes search-based algorithms (Dijkstra [14], A* [15], D* [16]) and sampling-based algorithms (RRT [17], RPM [18]). The artificial potential field method uses local environmental information obtained by airborne sensors to treat the robot environment as a potential field, in which the target point generates gravity to attract the robot and obstacles generate repulsion to repel the robot [19]. Velocity obstacle (VO)-based approaches [20,21] are widely used to predict collision regions and determine the robot’s velocity in real time. They incorporate the sensor information (e.g., laser scanners and cameras) within the control loop [22]. Gap-based methods have been proposed to determine subsequent actions in navigation tasks. Most reactive methods face the problems of falling into local minima and tending to oscillate in a narrow passage [3]. One can build a global environment map online, but it takes a lot of time to implement. 

With the rapid development of deep learning, the capabilities of feature extraction and function approximation have become more powerful. Combined with neural networks, high-dimensional state space information can be obtained [23]. Deep learning-based methods use deep neural networks to extract reasonable navigation behavior patterns from large amounts of labeled expert data. However, collecting labeled samples for navigation in unknown environments is time-consuming and energy-consuming, which hinders the widespread application of deep learning-based methods to solve the proposed problem [7]. DRL learns from labeled data, but its experience is generated from interactions between the agent and the environment, and thus it trains the neural networks with manually designed rewards [24,25]. The DRL approach transforms extensive training experience into the ability to think multiple steps ahead for more proactive movement decisions [21].

The deep reinforcement learning (DRL)-based collision avoidance approach can learn from a large amount of training experience, which is advantageous, and it can perform well in complex scenarios with high efficiency and robustness. Mnih et al. [26] applied a DRL algorithm to perform better than human players in a video game, in which the algorithm combined deep learning (DL) with reinforcement learning (RL) to solve complex decision-making problems. Based on the strong feature presentation ability of Q-learning (QL) [27] and convolutional neural network (CNN), deep Q-network (DQN) has shown its tremendous potential in robot control and decision making. Haarnoja et al. [28] developed the Soft Actor-Critic (SAC) algorithm to deal with inefficient exploration in continuous action settings. This model has been used with great success in control tasks such as dexterous hand manipulation [29] and DeepMind Control Suite [30]. In the field of robot control, the DRL methods in continuous action spaces can establish concise mapping from image inputs to the control policy [31]. Researchers have been applying DRL to navigate the intelligent agents in an unknown environment. Zeng et al. [7] proposed a novel DRL algorithm for continuously controllable navigation of non-holonomic robots in unknown dynamic environments with moving obstacles. A RL framework in decentralized collision avoidance was proposed in [32], in which each agent can independently make its decision without communicating with others. Zhelo et al. [33] proposed a curiosity-driven exploration strategy and discussed the robot’s ability to explore in complex and unknown environments. 

The state representation is critical for the performance of DRL, especially for real-time decision-making tasks of navigation in complex environments. Many previous works [7,34] have targeted RL models with vector-based state representations. Choi et al. [32] used the LiDAR data, the forward velocity (v) and the rotational velocity (ω) of the robot, and the relative distances of (x) and (y) from the robot to the target position to describe the robot’s state in the environment. Sparse LiDAR data is usually insufficient to describe the environment in detail, and in the case of any surrounding vehicles, it is necessary to find the interacting areas that have a more significant impact on the autonomous robot’s decision to make decision behavior safe and effective. Attentional mechanisms [35] have achieved great success in different areas, and self-attention is one of the most used approaches. The self-attention mechanism [36,37] uses a self-supervised approach to calculate the response at a certain position in the sequence. The attention mechanism can discover the inter-dependencies between a variable number of inputs and is suitable for autonomous driving decision making problems.

In this article, we proposed a state attention network (SAN) to extract features to represent the interaction state of an intelligent robot with its environment using a self-attentive mechanism. Based on the Soft Actor-Critic (SAC) algorithm, an auxiliary actor discriminator (AAD) was designed to evaluate for collisions before executing the action and guide the agent to explore the environment safely and improve exploration efficiency. Goal-directed and gap-based navigation strategies were proposed to guide robotic exploration and help the network converge faster. 

The structure of this paper is organized as follows: Section 2 introduces the framework of our approach and presents a method to train the neural network with AAD, goal-directed, and gap-based navigation strategies; Section 3 shows the simulation experiment and experimental result; and finally, Section 4 gives some conclusions about this paper.

## 2. Materials and Methods

### 2.1. Experiment Design and Data Collection

**Figure 1:**
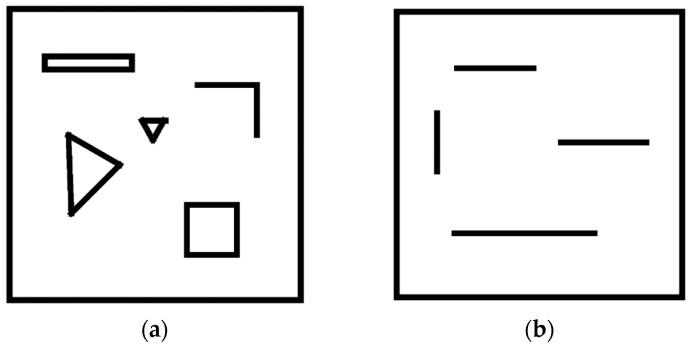
Two indoor environments (8 × 8 m^2^) were created, in which solid black lines were walls and hollow geometric figures were obstacles. (**a**) Environment I. (**b**) Environment II.

The virtual training environments were simulated by Gazebo, in which two indoor environments were constructed to demonstrate the effect of the environment on the model (Figure 1). A Turtlebot3 was applied as the robot platform to test the adaptability of the model in different environments. In our model, the control frequency is 5 Hz, and the moving steps of the robot are up to a maximum of 5000 in one episode. In every episode, the target position is randomly initialized throughout the area and is guaranteed not to collide with other obstacles.

The hyper-parameters of our method are described as follows: The learning rates for the critic and actor network are both 0.0001, the discount factor is 0.98, the goal tolerance distance is 0.15, and the replay buffer size is 1 × 10^6^. We trained the model with an Adam optimizer on a single Nvidia GeForce GTX 3070 GPU (i7-11700, RAM 16 G) for 1 × 10^8^ training steps which took almost 20 h.

The robot was tested to move from the given start point to the given end point without collision in an unknown environment. It sensed its own state and local environment state (*S*) through on-board sensors (Equation (1)). The inertial measurement unit calculates its own posture with the Euler angle description $\left[\theta_{r},\theta_{p},\theta_{y}\right]$, velocity information $\left[v_{x},v_{y},\omega_{z}\right]$, GPS estimates of the robot’s position $\left[p_{x},p_{y}\right]$, and LiDAR scans of the surrounding obstacles.

(1) $$S=\left[s_{t}^{lidar},s_{t}^{goal},s_{t}^{robot}\right]$$

(2) $$s_{t}^{goal}=\left[s_{goal},\theta_{goal}\right]$$

(3) $$s_{t}^{robot}=\left[p_{x},p_{y},v_{x},v_{y},\theta_{y}\right]$$

where $s_{t}^{lidar}$ are the LiDAR data which indicate the relationship of the obstacles and the robot by measuring the distance between the recognizing objects, $s_{t}^{goal}$ are the relative distance and heading angle from the robot to the target’s position, and $s_{t}^{robot}$ describe the state of the robot itself.

The action space is the robot’s linear velocity increment in the x-plane (Δ*v_x_*) and the angular velocity increment in the z-coordinate axis ($∆\omega_{z}$) (Equation (4)). The control velocity at the next time step (*v_t_*_+1_) should be the current velocity (*v_t_*) plus the robot’s action space with a constant *μ* (Equation (5)). The reward function (*r*) of RL model was set as Equation (6).

(4) $$a=\left[∆v_{x},∆\omega_{z}\right]$$

(5) $$v_{t+1}=v_{t}+\mu \cdot a,\;v_{t}\in \left[v_{min},v_{max}\right]$$

(6) $$r=\left\{\begin{array}{c} 500.0\;\;\;\;\;\;\;\;\;\;\;\;\;\;if\;d_{current}<0.2,\;d_{current}=dist\left(robot,goal\right)\\ -500.0\;\;\;\;\;\;\;\;\;\;\;\;\;\;\;\;\;\;\;\;\;\;\;\;\;\;\;\;\;\;\;\;\;\;\;\;\;\;\;\;\;\;\;\;\;\;\;\;\;\;\;\;\;\;\;\;\;\;\;\;\;\;\;\;if\;d_{obs}<0.2\\ -\lambda \cdot \frac{d_{current}}{d_{init}}\;\;\;\;\;\;\;\;\;\;\;\;\;\;\;\;\;\;\;\;\;\;\;\;\;\;\;\;\;\;\;\;\;\;\;\;\;\;\;\;\;\;\;\;\;\;\;\;\;\;\;\;\;\;\;\;\;other\;\;\;\;case\; \end{array}\right.$$

where *d_current_* indicates the distance of the current robot from the target point, *d_init_* is the initial distance, and *λ* is a factor that regulates the scale of the reward value.

### 2.2. Model Development

A modified SAC model was developed in this work. The SAC algorithm was developed to reduce inefficient sample sizes in continuous action settings [29], and it attempts to find a policy that maximizes the entropy objective (Equation (7)).

(7) $$\pi^{*}=\underset{\pi }{argmax}\sum_{t=0}^{T}E_{\left(s_{t},a_{t}\right)∼\tau_{\pi }}\left[\gamma^{t}\left(r\left(s_{t},a_{t}\right)+\alpha H\left(\pi \left(.|s_{t}\right)\right)\right]\right.\;r:S\times A\to R,\;\gamma \in \left[0,1\right],\;s_{t}\in S,\;a_{t}\in A$$

(8) $$H\left(\pi \left(.|s_{t}\right)\right)=-\log\pi \left(.|s_{t}\right)$$

where *π* is a policy; π* is the optimal policy; *T* is the number of timesteps (t); *r* is the reward function; *γ* is the discount rate falling; *s_t_* is the state at timestep *t*; $a_{t}$ is the action at timestep *t*; $\tau_{\pi }$ is the distribution of trajectories induced by policy *π*; *α* is the temperature parameter which is used to determine the relative importance of the entropy term versus the reward; and $H\left(\pi \left(.|s_{t}\right)\right)$ is the entropy of the policy *π* at state *s_t_* and was calculated in Equation (8).

The soft state value function (*V*) was applied to maximize the objective within the maximum entropy framework (Equation (9)). The soft q-function can be obtained by starting from a randomly initialized function $Q\left(s_{t},a_{t}\right)$ and repeatedly applying the modified Bellman backup operator ($T^{\pi }$) (Equation (10)). In the continuous state space, the soft q-function $Q_{\theta }\left(s_{t},a_{t}\right)$ was parameterized using a neural network with parameter *θ*. The soft q-function was trained to minimize the soft Bellman residual (Equation (11)).

(9) $$V\left(s_{t}\right):=E_{a_{t}∼\pi }\left[Q\left(s_{t},a_{t}\right)-\alpha \log\left(\pi \left(a_{t}|s_{t}\right)\right)\right]\;$$

(10) $$T^{\pi }Q\left(s_{t},a_{t}\right):=r\left(s_{t},a_{t}\right)+\gamma E_{s_{t+1}∼p\left(s_{t},a_{t}\right)}\left[V\left(s_{t+1}\right)\right]\;Q:S\times A\to R,\;p:S\times A\to S$$

(11) $$J_{Q}\left(\theta \right)=E_{\left(s_{t},a_{t}\right)∼D}\left[\frac{1}{2}{\left(Q_{\theta }\left(s_{t},a_{t}\right)-\left(r\left(s_{t},a_{t}\right)+\gamma E_{s_{t+1}∼p\left(s_{t},a_{t}\right)}\left[V_{\bar{\theta }}\left(s_{t+1}\right)\right]\right)\right)}^{2}\right]$$

where *p* gives the distribution over the next state when the current state and action have been given; $T^{\pi }$ is the modified Bellman backup operator; *θ* is the parameter of a neural network; *D* is the replay buffer of past experiences; and $V_{\bar{\theta }}\left(s_{t+1}\right)$ is an estimate of a target network of *Q*.

The soft q-function can guide the policy improvement step by updating the policy in a direction to maximize the obtained rewards. In the continuous state setting, the policy $\pi_{\phi }\left(a_{t}|s_{t}\right)$ was parameterized using a neural network with parameter ϕ, and output a mean and a covariance to define a Gaussian policy. The policy parameters ($\pi_{\mathrm{new}\;}$) were updated by minimizing the expected KL divergence ($D_{KL}$) using $\pi_{\mathrm{old}\;}$ (Equation (12)). Usually, the partition function $Z^{\pi_{\mathrm{old}\;}}\left(s_{t}\right)$ can be ignored since it does not impact the gradient.

(12) $$\pi_{\mathrm{new}\;}=\underset{\pi \in \Pi }{argmin}D_{KL}\left(\pi \left(.|s_{t}\right)∥\frac{\exp\left(\frac{1}{\alpha }Q^{\pi_{\mathrm{old}}}\left(s_{t},.\right)\right)}{Z^{\pi_{\mathrm{old}}}\left(s_{t}\right)}\right)$$

The output distribution of this strategy means that errors cannot be backpropagated in the normal way. To solve this, we used the reparameterization trick (Equation (13)) and obtained the new policy objective (Equation (15)).

(13) $$J_{\pi }(\phi )=E_{s_{t}∼D}\left[E_{a_{t}∼\pi_{\phi }}\left[\alpha log\left(\pi_{\phi }\left(a_{t}|s_{t}\right)\right)-Q_{\theta }\left(s_{t},a_{t}\right)\right]\right]$$

(14) $$a_{t}=f_{\phi }\left(\epsilon_{t};s_{t}\right),\;\epsilon_{t}\in N\left(0,1\right)$$

(15) $$J_{\pi }(\phi )=E_{s_{t}∼D,\epsilon_{t}∼N}\left[\alpha log\left(\pi_{\phi }\left(f_{\phi }\left(\epsilon_{t};s_{t}\right)|s_{t}\right)\right)-Q_{\theta }\left(s_{t},f_{\phi }\left(\epsilon_{t};s_{t}\right)\right)\right]$$

where $\pi_{\phi }$ is now defined implicitly in terms of $f_{\phi }$. Policy evaluation and policy improvement will converge to optimal policies. In continuous state space, the SAC algorithm has advantages in continuous state space. Therefore, the SAC algorithm was used as the base model in this paper.

**Figure 2:**
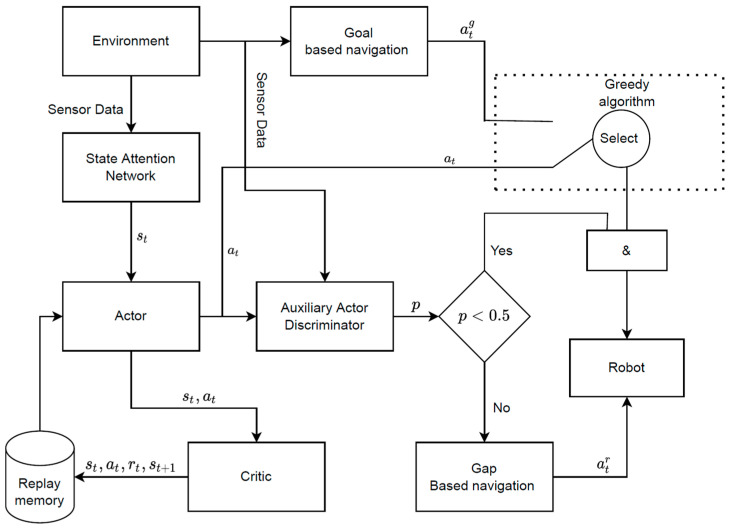
The modified SCA model. The actor was a neural network that can learn a navigation strategy from current state and make an action in real time, and the critic was a q-value function fitted using a neural network to evaluate state–action pairs. & means and.

The modified SAC model included a SAN for state learning, and an auxiliary action discriminator for collision probability calculation (Figure 2). It introduced prior knowledge to propose goal-based and gap-based navigation strategies to guide the learning strategies. Raw data from the LiDAR sensor and inertial measurement unit were aligned using a SAN based on an attention mechanism to a uniform state (*s_t_*). It is hard to prepare enough data to train neural networks when robots are exploring unknown environments. A replay memory D was applied to store experience (s, a, s′, r) to train the neural network by randomly sampling data. During the training phase, the robot moved randomly in unknown environments and generated a lot of collision data, which can make the model fall into local best. An AAD based on the RVO algorithm was developed to calculate the probability of a collision for the current action and reduce the number of collisions. With the help of prior knowledge, neural networks can quickly converge to an optimal action strategy.

### 2.3. State Attention Network (SAN)

**Figure 3:**
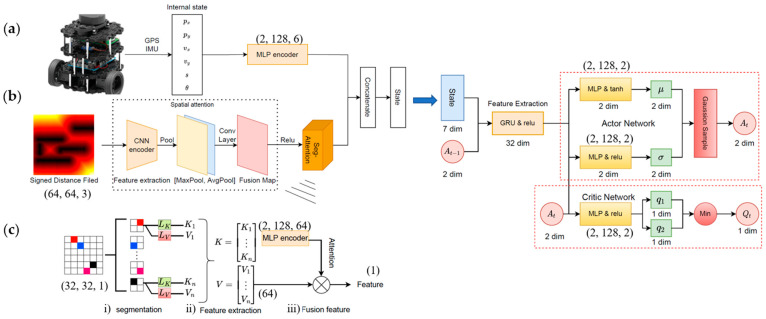
The proposed SAN’s structure: (**a**) robot’s information and (**b**) local environment information were analyzed to advance the model’s perceptual ability, in which (**c**) the seg-attention module structure extracted key information using a self-attention mechanism. The hidden layers, neuron number per layer, and output dimension of the MLP encoder are shown next to the network. The image size of the signed distance field is (64, 64, 3) and its output is (32, 32, 1). The final feature scale is (1) after the seg-attention module.

The state representation is critical in an autonomous driving task using DRL with multi-sensor data. For path planning tasks, the state space contains information related to the robot’s own state (e.g., speed, position, orientation angle) and the environment (e.g., obstacles). The input of the SAN includes the vector-based states and the image-based states. In this work, the vector-based states included the robot position, velocity, and distance to the target point. The orientation angle and the image-based states were represented by the LiDAR measurements using a signed distance field algorithm (Figure 3a).

The spatial attention module was proposed by CBAM to extract the weights of the spatial information, in which a CNN encoder was developed to extract image deep-level features. Two feature maps were obtained from maximum pooling and average pooling, and a deep-level fusion map was created through a convolution operation (Figure 3b). The segmented attention (seg-attention) module was developed to study correlations between different regions of the fusion map, which consisted of input segmentation, feature extraction, and fusion feature (Figure 3c). The input segmentation module segmented the fusion map into multiple partial states (small image patches in the grid). The feature extraction module extracted key (Equation (16)) and value (Equation (17)) features from each partial state to determine where the model should attend to encode information for path planning tasks.

(16) $$K_{i}=L_{k}*s_{i}$$

(17) $$V_{i}=f_{c}\left(L_{V}*s_{i}\right)$$

where *s_i_* is *i*-th partial state, *L_K_* and *L_V_* are the weight matrixes, *K_i_* is the key feature for *i*-th partial state, *V_i_* is the value feature for *i*-th partial state, and *f_c_* is the ReLU function.

The attention layers (neural network *θ*) determined important partial states based on key features (*K*) to obtain the attention weight vector (*A*) (Equation (18)). They were randomly initialized in the beginning of training. Value features were weighted by *A* (Equation (19)). After processing the image-based state, the robot’s own related state was encoded by the MLP and connected *f* to form a unified state space (Figure 3c).

(18) $$A=softmax\left(\theta *K\right)$$

(19) $$f=\sum_{i}V_{i}*A_{i}$$

### 2.4. Auxiliary Actor Discriminator (AAD)

**Figure 4:**
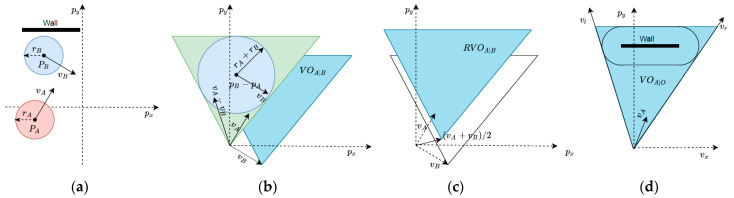
VO and RVO in a workspace configuration, where A and B are two moving robots in the 2D workspace ($p_{x},\;p_{y}$), $r_{A}$ and $r_{B}$ mean the radius that described the current state of robot A and B, $p_{A}$ and $p_{B}$ mean the quality hearts’ positions, $v_{A}$ and $v_{B}$ mean the current velocity of A and B, $VO_{A|B}$ means the VO area of the robot A generated by the robot B, and $RVO_{A|B}$ means the set of speeds that robot A can choose to be safe. (**a**) Workspace, (**b**) VO, (**c**) RVO, and (**d**) static line obstacle.

The velocity obstacle (VO) was developed for mobile robots to deal with dynamic obstacles in their local path planning. Robots *A* and *B* are obstacles to each other and both have independent target points. They both need to perform obstacle avoidance and plan a path to reach the target point (Figure 4a). The VO of *A* to *B* contains all possible collide velocities, and the VO area of the robot A generated by the robot B ($VO_{A|B}$) is a cone area with an apex at $v_{B}$ (Figure 4b) and can be calculated using Equations (20) and (21).

(20) $$A⨁B=\left\{a+b|a\in A,\;b\in B\right\},-A=\{-a|a\in A\}$$

(21) $$VO_{A|B}=\{v|\lambda \left(p_{A},v_{A}-v_{B}\right)\cap B⨁-A\neq \emptyset \}$$

where A⊕B is the Minkowski sum of two points sets in *A* and *B*, −*A* denotes the robot *A* reflects in its references point, and $\lambda \left(p,v\right)$ denotes the ray with a starting point of *p* and in the direction of *v*. In order to avoid collision with obstacles, robot A should choose a speed outside the VO area ($v_{A}\notin VO_{A|B}$). The reciprocal velocity obstacle (RVO) was calculated to reduce oscillations of the VO method (Equation (16)). The RVO was translated geometrically from $VO_{A|B}$ with a vector $\left(v_{A}-v_{B}\right)/2$, and an apex at $\left(v_{A}+v_{B}\right)/2$ (Figure 4c).

(22) $${RVO}_{A|B}=\{v|2v-v_{A}\in {VO}_{A|B}\}$$

An AAD was developed based on the RVO to calculate the probability of collision for the next action. The collision probability function ($P_{t}$) at time t represented the quality of the selected velocity $v_{t}$ judged by the joint RVO area (Equation (23)).

(23) $$P_{t}=\left\{\begin{array}{c} 0\;\;\;\;\;\;\;\;\;\;\;\;\;\;\;\;\;\;\;\;\;\;\;\;\;if\;\;v_{t}\notin {RVO}_{A|O}\\ {\left(\xi +1\right)}^{-1}\;\;\;\;\;\;\;\;\;\;if\;\;v_{t}\in {RVO}_{A|O} \end{array}\right.\;\left\{\begin{array}{c} v_{t}\notin {RVO}_{A|O}\;\;\;\;\;\;\;\;\;\;\;\;\;\;v_{t}\times v_{l}<0⋁v_{t}\times v_{r}>0\\ v_{t}\in {RVO}_{A|O}\;\;\;\;\;\;\;\;\;\;\;\;\;\;v_{t}\times v_{l}\geq 0⋀v_{t}\times v_{r}\leq 0 \end{array}\right.$$

where ξ is the estimated shortest time for the robot to collide with an obstacle at the current speed, and × is cross operate. After the action collision probability was calculated by the auxiliary action discriminator, the ε-greedy algorithm was applied to perform action selection (goal orientation or actor output) if no collision will occur. Otherwise, reactive navigation based on gaps was used to avoid obstacles and move towards the target point.

### 2.5. Prior Knowledge

It is important to have many valid data in the replay buffer to train the neural network effectively in learning strategies for navigation. In the traditional actor-critic algorithm, the robot randomly selected actions to explore, and the replay buffer stored many collision data, which were randomly selected to train the neural network. This can help the robot to learn collision avoidance strategies but may cause the robot to get stuck in a local solution, where the robot oscillates in a safe region and fails to achieve the effect of navigation. We used a priori knowledge and goal-directed/gap-based strategies (Algorithm 1) to guide the robot and reduce the network training time.
**Algorithm 1:** Our proposed algorithm
1:Initialize replay buff D, actor network $\pi_{\theta }$, old actor network parameters $\bar{\theta }\leftarrow \theta$, critic network $V_{\psi_{1}}$ and target critic network $V_{\psi_{2}}$, actor learning rate ${lr}_{a}$, critic learning rate ${lr}_{c}$, update frequency Κ, Collision threshold th, Temperature parameter α, target critic network update weight τ2:**for** episode = 1, N **do**3:  Initialize environment and set robot to the start point4:  **for** steps = 1, T **do**5:    Get Lidar data $l_{t}$, robot data $r_{t}$6:    Get uniform state $s_{t}$ using SAN module7:    Get current action $a_{t}$ by Actor Network8:    define final action $a_{t}^{*}$9:    Compute collision probability $p_{t}$ by AAD module.10:    **if** $p_{t}<\eta$ **then**11:     With probability ε select an action $a_{t}^{*}=a_{t}^{g}$ through goal-directed knowledge12:     With probability 1−ε select an action $a_{t}^{*}=a_{t}$13:    **else**14:     Get an action $a_{t}^{*}=a_{t}^{r}$ through Gap-based knowledge15:    **end if**16:    Execute action $a_{t}^{*}$, get reward $r_{t}$, and new state ${\overset{´}{s}}_{t}$17:    Store ($s_{t}$, $a_{t}^{*}$, $r_{t}$, ${\overset{´}{s}}_{t}$) in D18:    **if** steps%K==0 **then**19:     Randomly sample a minibatch ($s_{j},a_{j},r_{j},{\overset{´}{s}}_{j})\epsilon D$20:     Compute the target Q value:$$y_{target}=r_{j}+\gamma V_{\psi_{2}}\left({\overset{´}{s}}_{j},{\overset{´}{a}}_{j}\right)-\alpha \log(\pi_{\theta }({\overset{´}{a}}_{j}\left|{\overset{´}{s}}_{j}))\right.\;{\overset{´}{a}}_{j}\sim \pi_{\theta }({\overset{´}{s}}_{j})$$21:Update critic network parameters $\psi_{1}$ by minimizing the following loss function $$L_{V}^{loss}\left(\psi_{1}\right)=\frac{1}{2}\sum_{(s_{j},a_{j},r_{j},{\overset{´}{s}}_{j})\epsilon D}{\left(y_{target}-V_{\psi_{1}}\left(s_{j},s_{j}\right)\right)}^{2}$$22:Update actor network parameters θ by minimizing the following loss function $$L_{\pi }^{loss}\left(\theta \right)=\sum_{(s_{j},a_{j},r_{j},{\overset{´}{s}}_{j})\epsilon D}\left(\alpha \log\left(\pi_{\theta }\left(f_{\phi }\left(\epsilon_{j};s_{j}\right)\left|s_{j}\right.\right)\right)-V_{\psi_{1}}\left(s_{j},f_{\phi }\left(\epsilon_{j};s_{j}\right)\right)\right),\epsilon_{j}\sim N\left(0,1\right)$$23:Update temperature parameter α by minimizing the following loss function $$L_{\alpha }^{loss}\left(\alpha \right)=-\alpha \log\pi_{\theta }\left(a_{j}|s_{j}\right)+H_{0},H_{0}=-dim(A)$$24:Update target critic network parameters $$\psi_{2}=\tau \psi_{1}+(1-\tau )$$25:Update old actor network parameters $$\bar{\theta }\leftarrow \theta$$26:    **end if**27:  **end for**28:**end for**

The goal-directed knowledge is based on the angle ($yaw_{err}$) between the robot’s direction and the end point. The angle ($yaw_{err}$) is defined as the rotating angle from 0 to 360 degrees at which the robot rotates counterclockwise until it points to the end point. When the robot acquired the environment state data, the actor network gave the current action $a_{t}$. The probability of collision after executing this action was calculated by an auxiliary action discriminator, and an ε-greedy algorithm was used to select the final action to be executed between the goal-based $a_{t}^{g}$ (Equation (24)) and $a_{t}$ when the probability was less than a threshold η. With probability ε, the robot selected the action $a_{t}^{g}$ that would bring it closer to the end point according to the goal-directed knowledge.

(24) $$a_{t}^{g}=[0.05,\omega ]$$

(25) $$\omega =\left\{\begin{array}{c} yaw_{err}/180\;\;\;\;\;\;\;\;\;\;\;\;\;\;\;\;\;\;\;\;\;\;\;if\;yaw_{err}<180\\ 1-yaw_{err}/180\;\;\;\;\;\;\;\;\;\;\;\;\;\;\;\;\;\;\;\;\;\;\;\;\;\;\;othercase \end{array}\right.$$

To determine subsequent actions, the gap-based strategy analyzed the form of high-level descriptions of the environment on the basis of sensor information. The boundary gap model first extracted the gaps by analyzing the radar data to filter out the gaps that satisfied the passage conditions. The movement direction was selected according to the target point and the robot could be smoothly controlled through the gap.

## 3. Results

### 3.1. Radar Data Representation

**Figure 5:**
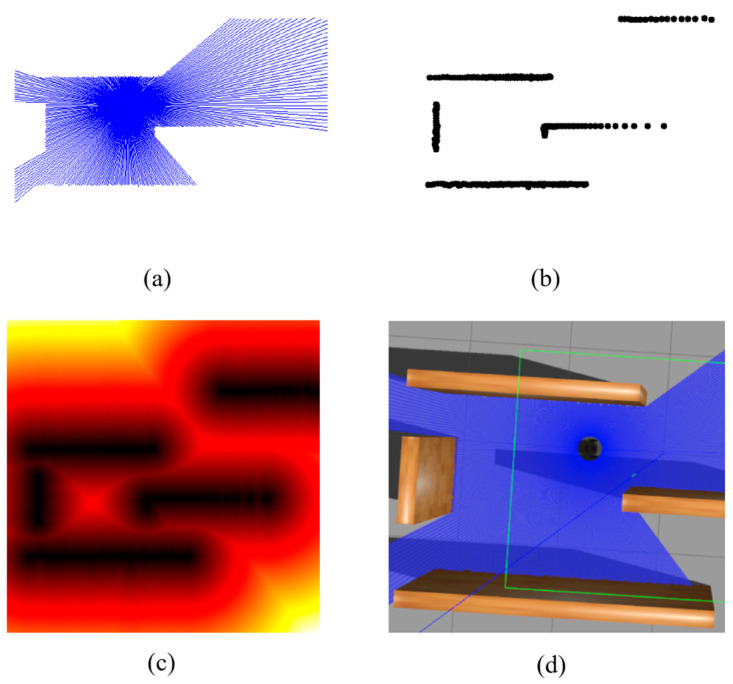
Graphical representation of radar data in process: (**a**) Represent the radar data in images, (**b**) extract the obstacle boundary, (**c**) calculate the distance obstacle map $s_{t}^{map}$ using the signed distance field algorithm, and (**d**) show in Gazebo environment. (**a**) Current LiDar data, (**b**) Extract obstacles, (**c**) Signed Distance Field, and (**d**) Gazebo environment.

Radar data was graphically represented to apply high-dimensional LiDAR data to RL model. The distance obstacle map $s_{t}^{map}$ was calculated using the signed distance field (SDF) algorithm (Figure 5). We use the $s_{t}^{map}$ as the description of the robot’s local environment.

**Figure 6:**
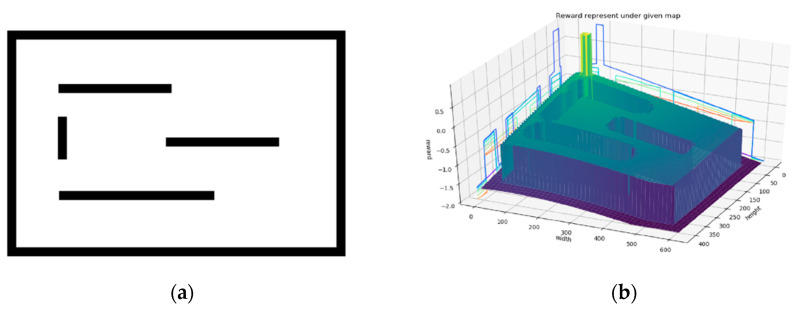
Reward function in Environment II. The start point is [3.0, −3.0]^T^, the end point is [−3.0, 3.0]^T^, λ = 100, and the reward value falls in [−2, 1]. (**a**) map, (**b**) reward.

The robot will keep moving closer to the target point while avoiding obstacles to get a higher reward. The reward function of Environment II is shown in Figure 6b.

### 3.2. Model Performance 

**Figure 7:**
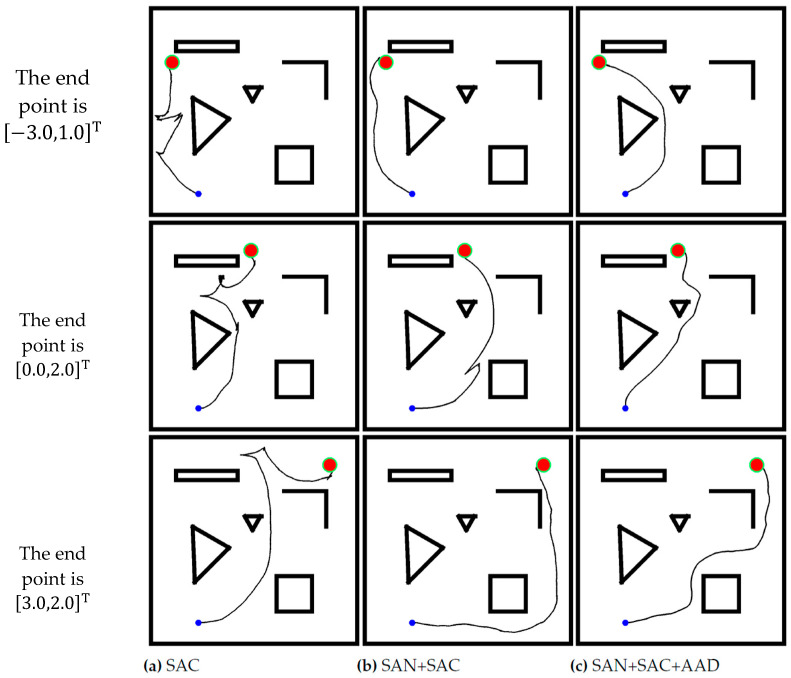
Nine trajectories that the robot obtained by using (**a**) SAC, (**b**) SAN+SAC, and (**c**) SAN+SAC+AAD models for three different end points in Environment I, in which the blue dot denotes the start point, the red circle in the upper right corner is the end point, and the black areas are four walls and obstacles.

The training phase and generalization of SAC, SAN+SAC, and SAN+SAC+AAD models were compared, and the parameters used in training were summarized in Table 1. In Environemnt I, the models were trained until the average reward of the smart car was stable, and the robot trajectories are shown in Figure 7. The figure shows that those three methods can guide the robot to the end point without collision. In general, the robot can move in the best trajectory in the SAN+SAC+AAD model. The robot can travel in a shorter path when it has learned heuristic knowledge.

**Figure 8:**
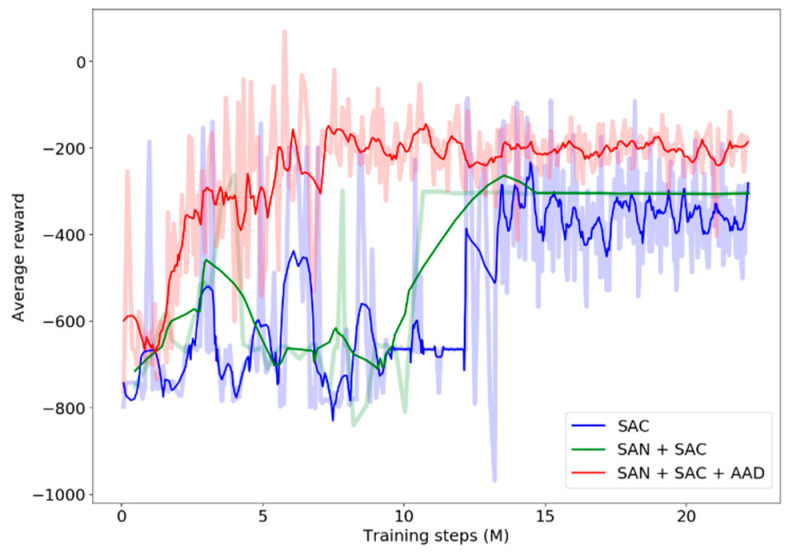
The average rewards of SAC, SAN+SAC, and SAN+SAC+AAD models in Environment I. Light lines mean the average rewards, and dark lines mean the values after smoothing.

The average reward of each model was calculated in an epoch (Figure 8). The average reward value fluctuates greatly in the early stage of training when we encourage robots to explore the map and expand the footprint range of the robot. SAC and SAN+SAC obtain similar average reward values in the convergence stage. Adding AAD, the number of collision obstacles reduced, and the highest average reward value was obtained in the early stage of training. 

**Figure 9:**
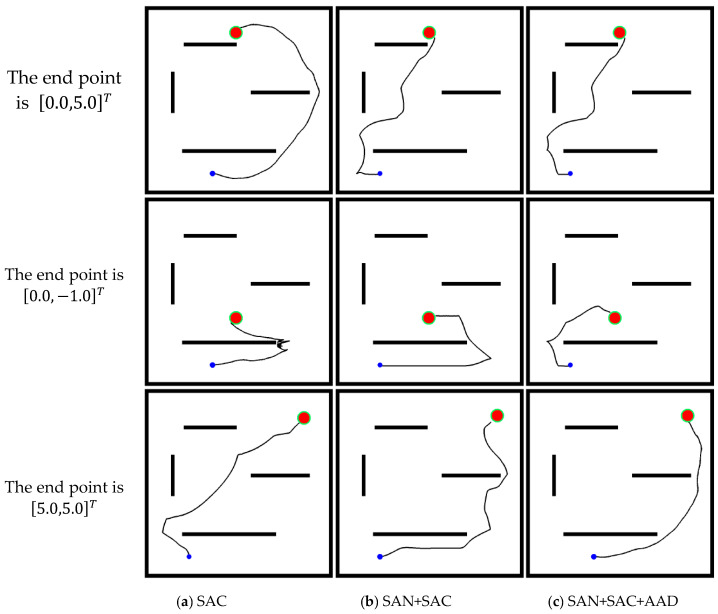
Nine trajectories that the robot obtained by using (**a**) SAC, (**b**) SAN+SAC, and (**c**) SAN+SAC+AAD models for three different end points in Environment II, in which the blue dot denotes the start point, the red circle in the upper right corner is the end point, and the black areas are four walls and obstacles.

The map was changed to Environment II to verify the model universality in different scenarios. The trajectories of the robot are shown in Figure 9. The obstacles in Environment II are relatively long. The robot chooses to bypass obstacles instead of looking for different topologies in SAC model. Adding SAN, the robot tries to find a better topological path. In the middle row, the target point ${\left[0,-1\right]}^{T}$ is close to the starting point ${\left[-2,-3\right]}^{T}$. The robot may keep away from the target point because of the obstacle direction. The robot needs to have a long-term vision and not be trapped in local minima. The robot oscillates constantly in the SAC model, but it has a better understanding of the environment in the SAN+SAC and SAN+SAC+ADD models.

**Figure 10:**
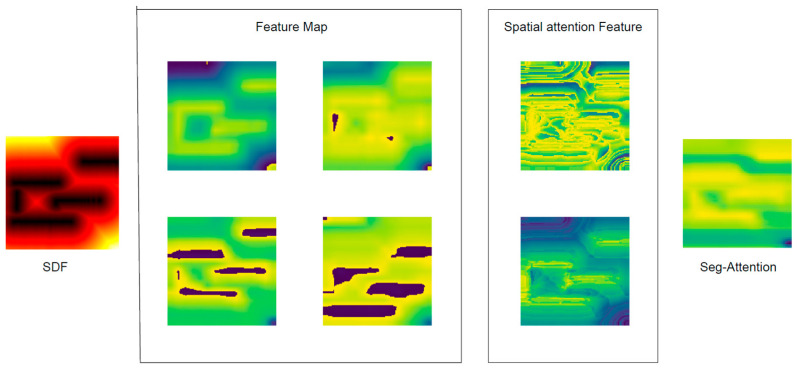
The visual feature map bypasses the SAN module, the SDF picture was used as the input of the SAN module, the feature maps were extracted using CNN, the feature maps were fused using spatial attention, and the seg-attention network was used to correlate the characteristics of different regions in the fused feature map, in which the blue areas mean the safe path and the yellow areas mean the obstacle wall.

The moving step count and average reward were calculated in Environment II (Table 2). The SAN module can enhance the model understanding of the environment and help the robots to find different topological paths. Adding SAN, the robot can reach the end point without hitting obstacles, and changing the obstacles and the environment cannot affect the robot’s performance (Figure 10). It shows the multi-heads’ attention can filter irrelevant areas, focus on obstacle walls, and find safe paths. The AAD module aims to find the shorter path to arrive at the target point. Adding AAD, the moving steps dropped and the average reward increased quickly.

The moving step count and the average training steps of the robot when it reaches the end point in Environment I and Environment II are listed in Table 3. The SAN module greatly reduces the average moving step counts and training steps, because SDF can provide more abundant environmental information for the model, and with CNN’s powerful reasoning ability, it can help the model to reach the target point faster. Compared to SAC, our method with SAN+SAC+AAD reduced training steps by 42.78% and 40.88% on each map.

### 3.3. Real Scenario Validation

**Figure 11:**
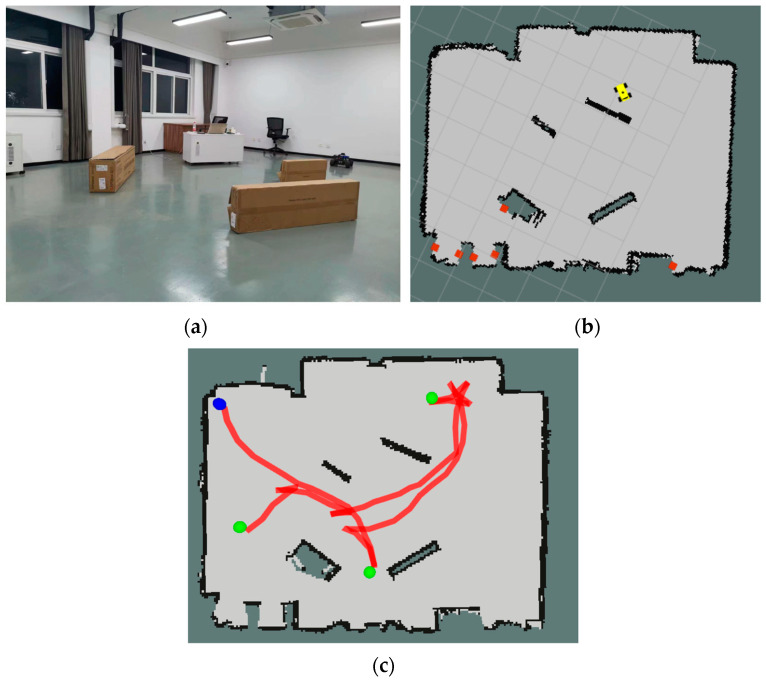
(**a**) Real scenario, (**b**) a map created using the Gmapping algorithm, (**c**) robot trajectory, in which the blue point is the starting point, and the green point is the target point.

In a real scenario, the RL algorithm collects robot data as the input to the model during the navigation process to decide the actions, including the robot velocity increments and the angular velocity increments. Commands are executed when the navigation task is completed or a collision occurs (Figure 11). On the outside, the mapping effect is poor because of the big LiDar measurement error and convex ground. The robot cannot actively explore because of the cumulative error.

**Figure 12:**
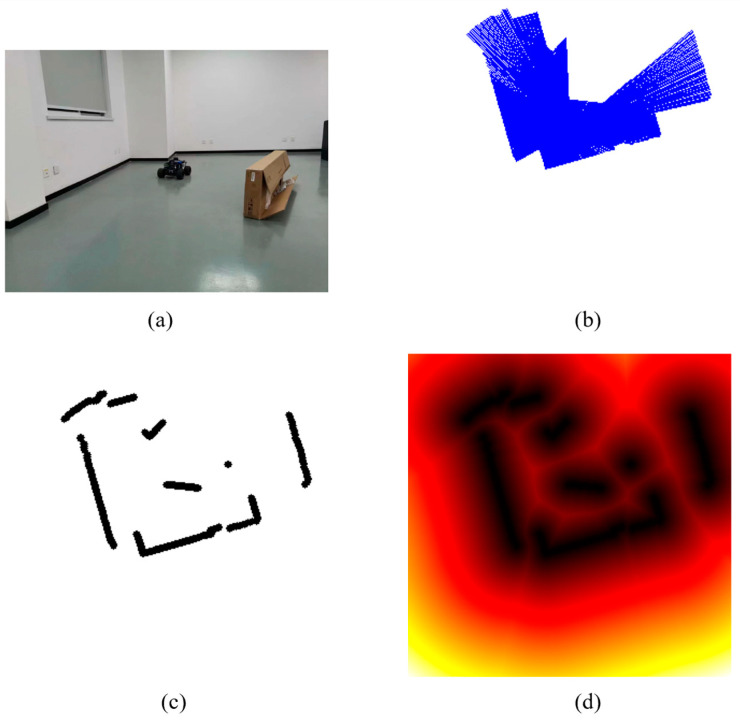
Visualization of LiDAR data using the SAN module in a real scenario. (**a**) Real Scene, (**b**) LiDAR data, (**c**) Obstacle Information, and (**d**) Signed Distance Field.

**Figure 13:**
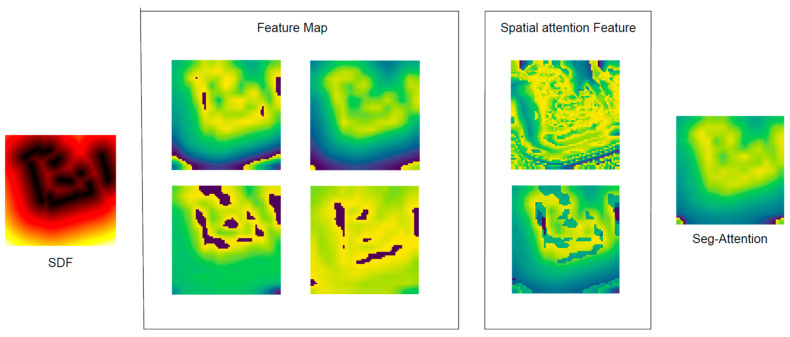
The visual feature map bypasses the SAN module in a real scenario, in which the blue areas mean the safe path and the yellow areas mean the obstacle wall.

Using the SAN module, the robot collected the LiDAR data and extracted the obstacle information to obtain SDF picture (Figure 12). The feature map focusses on safe paths and obstacles, and it makes good use of the distance information provided by the SDF map (Figure 13).

## 4. Conclusions

In this article, we combine SAC with a SAN module and an AAD module (SAN+SAC+ADD) for intelligent robots’ path planning and obstacle avoidance. The method has been tested in different environments and the results show that the robot is able to converge to the optimal policy faster and reach the end point in fewer steps than other methods. Experiments also show that our model has better adaptability in unknown environments. In this paper the obstacles are static. In the future, we will work on dynamic obstacle avoidance.

## Figures and Tables

**Table 1:** The values of training parameters.

Parameter	Value
Learning rate $l_{ra}$	0.001
Update frequency K	1 × 10^4^
Replay memory	1 × 10^6^
Collision threshold th	0.6
Temperature parameter α,τ	0.5
Mini-batch size	1024
Discount factor γ	0.99
Action selection factor ε	0.5

**Table 2:** Moving step count and average training steps in Environment II.

	Model	Target Point 1	Target Point 2	Target Point 3
Moving step count	SAC	1805	2049	2224
	SAN+SAC	1708	1921	2085
	SAN+SAC+ADD	**1573**	**1351**	**1558**
Average reward	SAC	−415.26	−524.36	−583.46
	SAN+SAC	−365.28	−460.87	−468.23
	SAN+SAC+ADD	−**294.57**	−**136.86**	−**281.39**

The numbers in bold mean the best model performance.

**Table 3:** Moving step count and average training steps.

	Model	Environment I	Environment II
Moving steps	SAC	3190	2594
	SAN+SAC	2714	1793
	SAN+SAC+AAD	**2106**	**1394**
Average train steps (M)	SAC	15.24	9.27
	SAN+SAC	12.68	8.26
	SAN+SAC+AAD	**8.72**	**5.48**

The numbers in bold mean the best model performance.

## Data Availability

Data are contained within the article. The code that supports the findings of this study is available from the corresponding author upon reasonable request.
